# Comparative Analysis and EST Mining Reveals High Degree of Conservation among Five *Brassicaceae* Species

**DOI:** 10.1155/2010/520238

**Published:** 2010-09-20

**Authors:** Jyotika Bhati, Humira Sonah, Tripta Jhang, Nagender Kumar Singh, Tilak Raj Sharma

**Affiliations:** Genoinformatics Laboratory, National Research Centre on Plant Biotechnology, Pusa Campus (IARI), New Delhi 110012, India

## Abstract

*Brassicaceae* is an important family of the plant kingdom which includes several plants of major economic importance. The *Brassica spp.* and *Arabidopsis* share much-conserved colinearity between their genomes which can be exploited for the genomic research in *Brassicaceae* crops. In this study, 131,286 ESTs of five *Brassicaceae* species were assembled into unigene contigs and compared with *Arabidopsis* gene indices. Almost all the unigenes of *Brassicaceae* species showed high similarities with *Arabidopsis* genes except those of *B. napus,* where 90% of unigenes were found similar. A total of 9,699 SSRs were identified in the unigenes. PCR primers were designed based on this information and amplified across species for validation. Functional annotation of unigenes showed that the majority of the genes are present in metabolism and energy functional classes. It is expected that comparative genome analysis between *Arabidopsis* and related crop species will expedite research in the more complex *Brassica* genomes. This would be helpful for genomics as well as evolutionary studies, and DNA markers developed can be used for mapping, tagging, and cloning of important genes in *Brassicaceae*.

## 1. Introduction

*Brassicaceae* species consisting of various agronomically important crops like oilseeds, broccoli, cabbage, black mustard, and other leafy vegetables are cultivated in most parts of the world. The genus *Brassica *is evolutionarily closely related to model crucifer plant *Arabidopsis thaliana*, since both are members of the family *Brassicaceae* and reported to have diverged 14–20 million years ago [1]. The major centers of diversity of *Brassicaceae* family are southwestern and central Asia and the Mediterranean region whereas the arctic, western North America, and the mountains of South America are secondary centers of diversity [2]. The genus *Brassica* is a monophyletic group within the *Brassicaceae*. It includes the cultivated oil seeded species, *Brassica juncea*, *B. napus,* and *B. rapa *and vegetable *B. oleracea*, which are also very closely related to *A. thaliana*. The genomes of the three diploid *Brassica* species, that is, *B. rapa*, *B. nigra,* and *B. oleracea*, have been designated as A, B, and C, respectively, where as the genomes of the amphidiploids, *B. juncea* and *B. napus*, have been designated as AB and AC, respectively [3–5].

Comparative genomics is a powerful tool for genome analysis and annotation. There are two basic objectives for comparative genomics. First, to understand the detailed process of evolution at the gross level (the origin of the major classes of organism) and at a local level (what makes related species unique) [6]. Second, to translate DNA sequence data into proteins of known functions. The rationale here is that DNA sequences encoding important cellular functions are more likely to be conserved between species than sequences encoding dispensable functions or noncoding sequences. 

The biology of *Arabidopsis* and *Brassica* are very similar. However, because of polyploidy nature of *Brassicaceae* species, their genomes are more complex compared to *A. thaliana*. *A*. *thaliana* serves as a model for comparative microsynteny studies with *Brassica* species because of its small genome (with less repetitive DNA), short generation time, and well-established genetic and genomics resources [7]. A pattern of chromosomal colinearity has been identified between *Arabidopsis* and *Brassica* plants [7]. Since the *Brassica* and *Arabidopsis* belong to the same *Brassicaceae* family, the level of synteny between them may provide a good opportunity to study how genetic and morphological variation has developed during the evolution of the genome, including the endurance of certain genetic structures in *Arabidopsis* and related *Brassica* species [7]. Hence, comparative genome analysis may lead to a better understanding of plant of closely related species.

ESTs are considered as important genomic resources for mining DNA markers based on simple sequence repeats (SSRs). The SSRs are present and distributed in the genomes of all eukaryotes. Because of the abundance and specificity of SSRs, these are considered as important DNA markers for genetic mapping and population studies. The important features of SSR markers coupled with their ease of detection have made them useful molecular marker in different crops [8]. Therefore, detection of SSRs in the unigenes and ESTs of *Brassicaceae* species may help in designing a new set of DNA markers and may provide more insight in the evolution of these species. Once validated, these markers can be used by the breeders in different *Brassica *improvement programmes.

The analysis of GC contents among unigenes and ESTs gives important indication about the gene and genome compositions. The GC content of the sequence gives a fair indication of the melting temperature (*T*_*m*_) and stability of the DNA molecules. The positive correlation has been obtained with the higher GC content and absolute values of thermostability, bendability, and ability to B–Z transition of DNA structure whereas negative correlation has been obtained between the curvature and high GC content of the DNA molecule. The GC-rich DNA constitutes gene-rich, actively transcribed genomic regions hence considered good as functional or expressed DNA [9]. The GC content of sequences surrounding to the gene(s) also considered as the best predictor of the rates of substitution during evolution [10]. However, such analysis is lacking in case of different *Brassica* species.

In this study, the gene indices were constructed and comparative analysis for five *Brassicaceae* species, namely*, B. juncea*, *B. napus*, *B. oleracea*, *B. rapa,* and *R. sativus* was reported for the first time. These gene indices constitute a total of 131,286 nonredundant sequences which was utilized to assess sequence conservation among *Brassicaceae* on a genomic scale, mining SSRs, frequency and type of repeat elements, and finding GC contents. DNA markers were designed and validated across *Brassica* species using PCR. Using the computational method, we have identified sequence and functional similarity of *Brassicaceae* transcripts to that of *Arabidopsis*, suggesting that a portion of these transcripts have a high degree of conservation with *Arabidopsis* genome. These analyses provide insight into the overall sequence conservation among *Arabidopsis* and *Brassicaceae* and within *Brassicaceae*.

## 2. Materials and Methods

### 2.1. Clustering of ESTs of *Brassicaceae* Species

For this study, a total of 131,286 ESTs deposited till August 2006 in the public database NCBI (http://www.ncbi.nlm.nih.gov/) representing the *Brassicaceae* species; *B. juncea* (235), *B. napus* (88,573), *B. oleracea* (20,923), *B. rapa* (21,422), and *R. sativus* (133) were downloaded. The available ESTs of these species were clustered into gene indices that represent a nonredundant set of transcripts or unigenes. Batch files of EST sequences for these species were downloaded in FASTA format. The sequences were clustered by using the SeqMan programme of DNASTAR software (http://www.dnastar.com/) to eliminate redundancies and generate unigene sequences. For clustering, we optimized clustering parameters in DNA Star software by using sample data created by taking random sequences of known genes. The optimized parameters were found to be efficient to cluster ESTs to a specific expected cluster and did not produce false joins among the ESTs.

### 2.2. Analysis of GC Content and SSR

The GC content of all the five *Brassicaceae* species was calculated using the formulae in excel sheet. We calculated the number of G and C separately, summing the two quantities and dividing by the total number of bases in that unigene sequence and then computing the percentage of GC contents.

The unigene sequences were used to identify SSRs using MISA software (http://pgrc.ipk-gatersleben.de/misa/). Six classes of SSRs, that is, mono-, di-, tri-, tetra-, penta-, and hexanucleotide repeats were targeted for identification using this tool. The default setting used in the program for minimum number of repeats was 10 for mononucleotide, 6 for dinucleotide, and 5 for tri-, tetra-, penta-, and hexanucleotides. In addition, this program also identifies complex repeats. Batch files of the target species were exported to the local database in Sun server using FTP and were run through MISA by passing the sequence file as input to the program at the command prompt. The output files were transferred to desktop using FTP and opened using excel sheets for visualizing the results. The four classes of mononucleotide SSRs were defined based on the repeat length, that is, mononucleotides 15 or less bp, 16–30, 31–45, and 46 or more bp repeats. The class chosen for dinucleotide repeats were 5–10 bp repeats, 11–16 bp repeats, and 17 or more bp repeats, while that for trinucleotide repeats were 5–10 and 11–16 bp. Results on repeat types, number of repeats, and frequency across all species were tabulated and significant results and observations were depicted in the form of different figures.

### 2.3. Functional Annotation of Unigenes

The unigene sequences of the five *Brassicaceae* species were matched with *Arabidopsis* gene sequence database at local BLAST server using BLASTN (with advanced options: -G5, -E1, -q1, -r1, -v1, and -b1). The results were extracted using in-house developed Perl scripts, and tabulated in excel sheet. The *Arabidopsis* unigene set was used as a reference, and the sequences of each of the five crops were split into batches of 200 each for comparisons. The results were tabulated and the bit score cutoff of 100 was applied to filter significant matches. These sieved hits were then BLAST searched against nr database using BLASTX (http://blast.ncbi.nlm.nih.gov/Blast.cgi) for annotation. The annotated genes were classified into 28 different functional categories based on their homology to known proteins.

### 2.4. Validation of SSR Markers

Five different species of Brassica, namely, B. ra*pa*, *B. carinata*, *B. juncea*, *B. napus,* and *B. oleracea* as well as *R. sativa* were used in the present study. All the species were subdivided into 2 to 3 groups (Table 1). Total genomic DNA was extracted from the fresh leaves of all Brassica species using CTAB method. Thirty four Genomic SSR markers, 15 unigene-derived and 39 genomic survey sequences (GSS) SSR were used to study their transferability across the species. The polymerase chain reaction (PCR) conditions, particularly annealing temperature for each primer, were standardized using gradient temperature ranges from 50°C to 60°C. The PCR reactions were performed using PTC 225 gradient cycler (BIO-RAD Inc.) in 10 *μ*L volumes containing 30 ng of brassica genomic DNA, 5 pmole, each of the forward and reverse primers, 0.1 mM dNTPs, 1x PCR buffer (10 mM Tris, pH 8.0, 50 mM KCl and 50 mM ammonium sulphate), 1.8 mM MgCl_2_, and 0.2 unit of *Taq *DNA polymerase. The PCR cycling conditions involved initial DNA denaturation at 94°C for 5 min followed by 30 cycles of denaturation at 94°C for 1 min primer annealing at 55°C–60°C for 1 min and primer extension at 72°C for 1 min. This was followed by a final extension step at 72°C for 10 min followed by storage at 4.0°C. The amplified products were resolved on 3% agarose gel using 1x TBE buffer, run at 120 V for 2 to 3 h depending on the size of the expected PCR product, and visualized using ethidium bromide staining using GEL documentation system. The band sizing of the amplicon generated by each SSR marker was determined as against 100 bp DNA ladder.

## 3. Results

### 3.1. Clustering of ESTs into Unigenes

A total of 131,286 EST sequences for five different crucifer family members were downloaded from the GenBank including dbESTs. These ESTs were generated from different tissues and stress levels by various workers (http://www.ncbi.nlm.nih.gov/). All sequences for each species were clustered into 25,428 unigenes (http://203.122.19.19/plantgenomedb/plantgenomedb.html) in five species. Less-abundant or lowly expressed transcripts could not be assembled into larger contigs remained as singletons. A summary of the EST and unigenes of each species is given in Table 2. In case of *B. juncea,* 83.4% of EST formed unigenes followed by *B. oleracea* (49.14%), *B. rapa* (41.14%), and *B. napus* (6.82%). We found only 133 EST sequences in case of *R. sativus* of these 70.68% formed 94 unigenes.

### 3.2. Similarity of *Brassicaceae* Gene Indices with Arabidopsis Genes

Using *Arabidopsis* gene indices, a comparative analysis of *Arabidopsis* with the five *Brassicaceae* species gene indices exhibited high level of similarity with the unigenes of *B. juncea*, *B. napus*, *B. oleracea*, *B. rapa,* and *R. sativus* (Table 3). The analysis based on EST-derived unigenes in these five *Brassica* species revealed that the majority of the gene indices have very less sequence variation compared to *Arabidopsis* gene indices and are conserved across the *Brassicaceae* family.

### 3.3. Analysis of GC Content of *Brassicaceae* Unigenes

**Figure 1:**
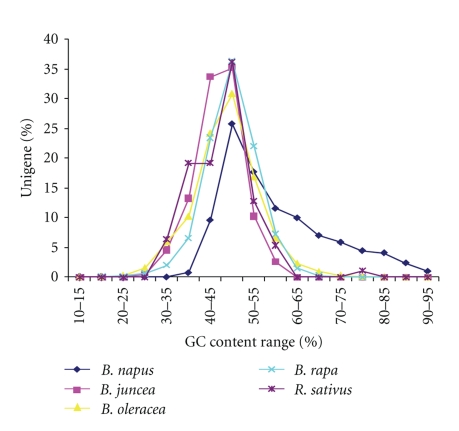
Frequency distribution of Unigenes with respect to GC content in five brassica species The average GC content of all the species was between 50%–55% and symmetrical in distribution except for *B. napus *which showed skewed distribution ranging from 30%–95%.

We analyzed the GC content (ratio of guanine and cytosine) of all the unigenes, and results were tabulated based on the class intervals defined in the range from 10%–95% GC content, with an interval of 5%. The GC content range of the transcripts of all the unigenes of 5 *Brassicaceae* species is given in Figure 1. The average GC content of all the species was between 50%–55% and symmetrical in distribution except for *B. napus *which showed skewed distribution ranging from 30%–95%. The GC content of *R. sativus* unigenes was quite variable (Figure 1).

### 3.4. Distribution of Repeat Length Classes in Unigenes

We found that in all the five *Brassicaceae* species explored in present study, most of the unigenes contained a single SSR stretch from which potential unique markers can be derived. The frequency of single SSR-containing unigene ranged from 60% (*B. rapa*) to 92% (*R. sativus*). The average frequency of unigenes containing multiple SSRs across all five species was 25%. The maximum number of unigene containing single SSR was found in case of *B. rapa*, followed by *B. juncea* and *B. oleracea *(Table 4). The SSR frequency observed was not uniform among these *Brassica *species (*x* ^2^ = 456.2, *d* *f* = 4). The relative abundance of mono-, di-, tri-, tetra-, penta-, and hexanucleotide repeats in all the five *Brassicaceae* species were determined by calculating their frequencies in the unigenes. The mononucleotide repeats were predominant in all the five species studied in present investigation. The frequency of mononucleotide repeats varied from 60% in *B. rapa* to 92% in *R. sativus*. The second dominant class was dinucleotide repeat in all species except *B. juncea*, which had trinucleotide repeat at second position. In rest of the species, highest percentage of mononucleotide repeats were obtained followed by di-, tri- and tetranucleotide repeats. A little variation observed at penta- and hexanucleotide where frequency of hexanucleotide was greater than pentanucleotide repeats.

### 3.5. Frequencies of Different SSR Repeat Types

**Figure 2:**
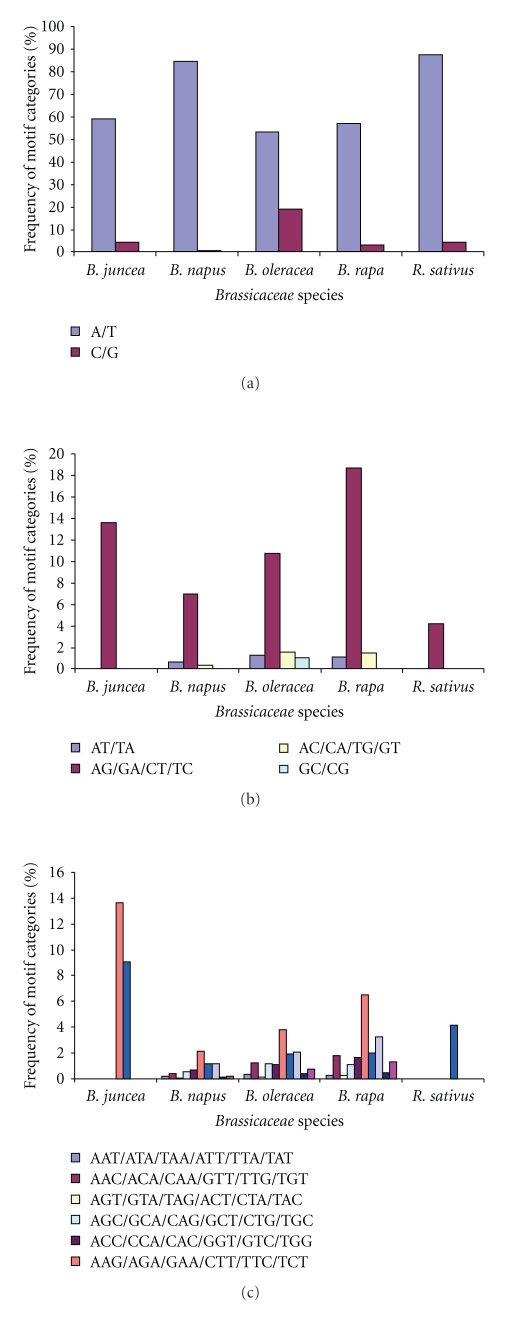
Frequency of SSR motif types in the unigenes. (a) mono-, (b) di-, and (c) trinucleotide repeats in unigene sequences signifying uneven distribution of different motifs in five *Brassica* species.

The relative frequencies of SSRs were calculated for five species. The frequency estimates shown are based on the total number of SSRs observed in all unigenes that have either single or multiple SSRs. It was seen that A/T repeats were the predominant mononucleotides in all the five species. The results indicated that A/T SSRs represent more than 50% of the total SSRs in all five species whereas the frequency of C/G repeats were 19.14% in *B. oleracea*, 4.55% in *B. juncea,* and 4.17% in *R. sativus *(Figure 2(a)). Among dinucleotide SSRs, AG/GA/CT/TC group was a ruling class of dinucleotide repeats in all of the species analyzed during this investigation. It ranged from 4.2% to 18.7% of the total SSRs explored. These repeats were maximum in *B. rapa* followed by *B. juncea*, *B. oleracea*, *B. napus,* and *R. sativus*. The average frequency of AT/TA and AC/CA/TG/GT was almost same (0.61% and 0.67%, resp.) among the five species (Figure 2(b)).

An assay of frequencies of trinucleotide repeats of total SSRs showed the predominance of AAG/AGA/GAA/CTT/TTC/TCT repeats class in 4 out of 5 species. For instance, the trinucleotide repeats were 22.73% in *B. juncea*, 18.48% in *B. rapa*, 12.85% in *B. oleracea*, 6.62% in *B. napus,* and 4.17% in *R. sativus *(Figure 2(c)). In *R. sativus,* the only ATG/TGA/GAT/CAT/ATC/TCA repeat class was found, which is the second dominant class of repeats in *B. juncea*. The AGG/GGA/GAG/CCT/CTC/TCC repeat was the second dominant class in *B. napus, B. oleracea, and B. rapa*.

The possibility of tetranucleotide repeats is 33 across the genomes [11, 12], but only a small number of tetra nucleotide repeats were observed among the 5 *Brasisca* species in present study. As the numbers are too low for frequency evaluation, all of the observed tetranucleotide repeats were assayed in order to figure out the most recurrent tetranucleotide SSRs across these *Brassicaceae* species. The top 15 tetranucleotide repeats obtained in the 5 *Brassicaceae* species were AAAC, AAAG, ATGA, CCAA, CTTT, GAAC, TACA, GAAA, AGAA, TTGT, TCAA, TTTG, AATC, CAAA, and GAAG. The AAAC and AGAA repeats were the most abundant tetranucleotide SSRs.

### 3.6. Frequencies of Different SSRs Repeat Length Classes

**Figure 3:**
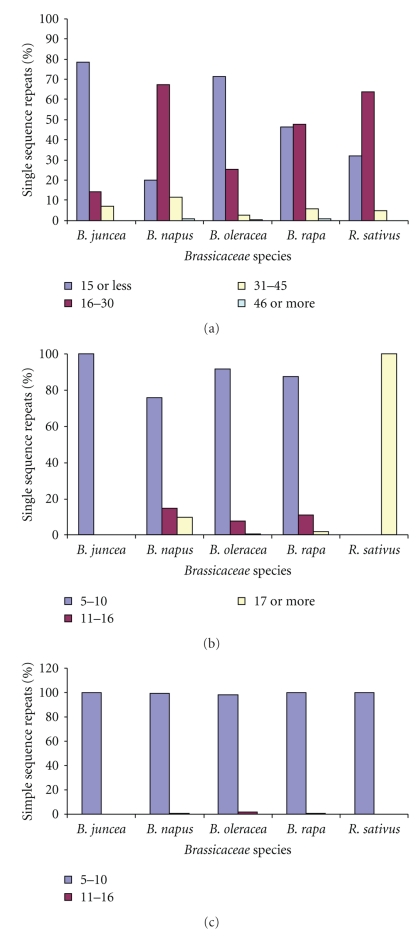
Relationship between different motif types of SSRs. (a) mono-, (b) di-, and (c) trinucleotide repeats and repeat length observed in unigene-derived SSRs of five *Brassica* species.

It was found that the majority of mononucleotide SSRs fall in 16–30 repeat classes followed by 15 or less repeat classes, except in *B. juncea *and *B. oleracea,* where 15 or less repeat classes were more abundant than 16–30 repeat classes (Figure 3(a)). In *B. rapa,* the 15 or less and 16–30 repeat classes almost shared nearly equal distribution of the SSRs. Although SSRs with 46 or more repeats were less frequent in all species. Distribution of dinucleotide SSRs showed that in most of species, they fall in the category of 5–10 repeat classes succeeded by 11–16 repeat classes (Figure 3(b)). However, in *R. sativus, *SSRs were detected in 17 or more repeat classes. With respect to the occurrence of trinucleotide SSR distribution into repeat length classes, the 5–10 repeat classes were most predominant in all the species analyzed (Figure 3(c)). Thus, the distribution of SSRs clearly showed the predominance of mononucleotide SSRs containing 16–30 repeats and di- and trinucleotide containing 5–10 repeats.

### 3.7. Functional Annotation of the Unigenes

The data from the completely sequenced *Arabidopsis* genome was used to predict genes and use them to compare with other species. The unigene sequences from five *Brassicaceae* species; namely,* B. juncea*, *B. napus*, *B. oleracea*, *B. rapa,* and *R. sativus *were used in this analysis. The functional categories of different unigenes are given in Figure 5. The most predominant functional category of unigenes were metabolism and energy, consisting of 33.71% of the total unigenes in *B. juncea* and 25.32% in *R. sativus* followed by *B. napus* (21.15%), *B. rapa* (20.72%), and *B. oleracea* (19.48%). The second dominant functional category was structural/catalytic proteins, which consisted of 15.41% of the total unigenes in* B. rapa*, followed by 13.92% in *R. sativus*, 13.52% in *B. oleracea*, 10.07% in *B. napus,* and 8.99% of the total unigene in *B. juncea*. Few other dominant functional categories were cell localization, protein activity regulation, and cellular transport (Figure 5). In two *Brassicaceae* species, that is, *B. juncea* and *R. sativus* common functional categories like cellular communication/signal transduction, interaction with environment (systemic), transposable elements, viral and plasmid proteins, cell type differentiation, organ differentiation, subcellular localization, organ localization, and nuclear protein were not obtained (supplementary Table 1 available online at doi:10.1155/2010/520238).

### 3.8. Validation of SSR Markers

**Figure 4:**
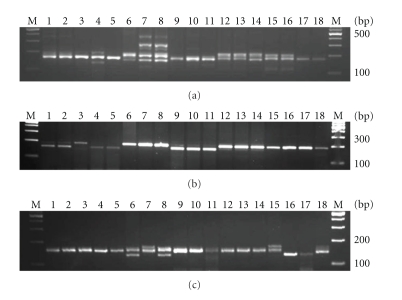
Amplification profile of (a) genomic SSR marker Bo_Genomic 90, (b) unigene SSR marker U_boleracea_506 (c) GSS-SSR marker GSS_Bn_464 in 18 genotypes belonging to Brassica species, lane 1, 2, 3* B. rapa toria*, lane 4, 5 *B. rapa* cv Yellow sarson, lane 6, 7, 8 *B. carinata, *lane 9, 10, 11 *B. juncea, *lane 12, 13, 14 *B. napus,* lane 15, 16, 17 *B. oleracea, *lane 18 *Raphanus sativa*.

**Figure 5:**
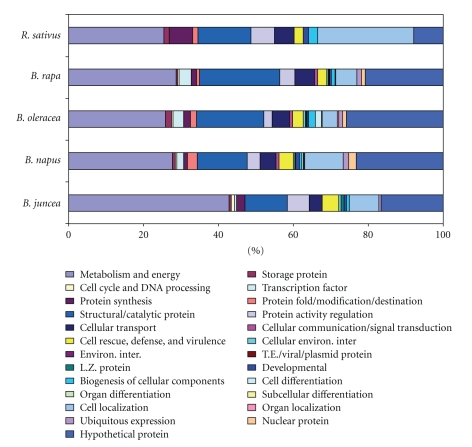
Frequency of genes in different functional categories analysed in five Brassicaceae species. The predominant functional category of unigenes was metabolism and energy followed by structural/catalytic protein. Most of the unigenes of all the species were hypothetical in nature.

To determine amplification efficiency of SSR markers, 35 genomic, 39 GSS and, 15 unigene-derived markers were chosen and used in PCR amplification. Thirty-one (88.57%) of the 35 genomic SSR markers, thirty-two (82.05%) of 39 GSS-SSR, and fourteen (93.3%) of 15 unigene SSR were successfully amplified (Table 5). Most of the markers produced fragments of expected size. The number of alleles amplified per locus ranged from 1 to 5 for genomic SSR, 1 to 2 alleles in case of unigene SSR and from 1 to 3 alleles in case of GSS-SSR (Figure 4). All the markers amplified similar as well as different size of DNA fragments in case of *Brassica* spp. Most of the primers were showing polymorphism within and between *Brassica* species. Genomic SSR showed 63% polymorphism, unigene-derived SSR showed 40% whereas GSS-SSR showed 86% polymorphism across all brassica genotypes analyzed in this study. Our study thus identified markers that are cross-transferable among different *Brassica species*.

## 4. Discussion

Crops belonging to *Brassicaceae* family are closely related to *Arabidopsis thaliana*. Since the whole sequence of *A. thaliana* genome has been decoded and is in public domain [13], it can be effectively used in comparative genome analysis with the genomic sequence of *Brassica *species to understand biological processes and manipulating different traits. In the present investigation, a comprehensive and detailed analysis of *Brassicaceae* unigenes was made and compared with that of *A. thaliana* gene indices. Our analysis showed that *Brassica* and *Arabidopsis* genes share high percentage of sequence identity hence can be used in various functional genomic studies in *Brassicaceae*. 

Analysis of GC contents showed that the unigenes of *B. juncea*, a tetraploid species have more GC content than another tetraploid species like *B. napus*. Even the unigenes of *B. napus* were less than that of diploid species *B. oleracea* and *B. rapa* [14]. It has also been reported that the GC contents may vary even in phylogenetically related species like onion and rice [14]. In other studies the mean GC content of coding regions is higher in angiosperms compared to the dicots [15]. However, from present investigation, such conclusions cannot be drawn since we have taken all the unigene sequences and did not distinguish among coding or noncoding regions. A gradient in GC contents along the direction of transcription has been obtained in case of *gramineae* genes [16]. Their exhaustive analysis showed that 5′-ends of *gramineae* genes were having 25% higher GC contents than their 3′-ends. Similarly, microsynteny analysis between *Oryza sativa* spp *japonica* and *O. sativa* spp. *indica* showed presence of higher average GC contents in *japonica* genes than in the *indica* genes [17].

The frequencies of different classes and types of SSRs have been calculated in the unigenes of five species within *Brassicaceae* species. Simple sequence repeats are found to be in abundance and consistently distributed in plant genomes. It has also been reported that SSRs occur as frequently as once in about 6 kb in case of plant genomes [18]. SSRs are more common in the vicinity of genes than in other regions of the genome [19]. However, among five *Brassicaceae* crops studied in present investigation, 62.45% of the unigenes of *B. napus* contained SSRs. 

Theoretically, the probability of finding mononucleotide repeats in a genome is higher followed by dinucleotide repeats and then by trinucleotide repeats followed by tetra-, penta-, and hexanucleotide repeats [20]. This trend of distribution of repeats for all the species, namely*, B. napus, B. oleracea, B. rapa, and R. sativus* has also been found in present study. However, the trinucleotide repeats were the second abundant in *B. juncea*. The frequency of hexanucleotide repeats found in *B. napus, B. oleracea, and B. rapa *is more than that of pentanucleotide repeats. The general trend showed that mononucleotides were the most abundant repeats in all five species followed by di- and trinucleotide repeats.

The available SSR motif combination could be grouped into unique classes based on the property of DNA-based complementarities. For mononucleotides, although A, T, C, and G are possible, A and T could be grouped into one category since an A repeat on one strand is same as a T repeat on the opposite strand and a poly C on one strand is the same as a poly G on the opposite strand, resulting in two unique classes of mononucleotides, A/T and C/G [11]. Similarly, in our study, all dinucleotides can be grouped into four unique classes: (i) AT/TA; (ii) AG/GA/CT/TC; (iii) AC/CA/TG/GT and (iv) GC/CG. Thus, the number of unique classes possible for mono-, di-, tri-, and tetranucleotide repeats is 2, 4, 10, and 33, respectively, [11, 12]. Major role of repeat elements has been attributed to the gene duplication and amplification for generating new alleles in a population. The whole genome analysis of rice and *Arabidopsis* has shown very interesting observations. In whole rice genome, a total of 18,828 classes of di-, tri-, and tetranucleotide SSRs representing 47 distinct motif families have been annotated [21]. It has been reported that 51 hypervariable SSR per Mb of the rice genome are available. These SSRs also used as DNA markers for specific regions of the genome, amplified well with PCR, polymorphic among different genotypes thus are of immense applications in genetic analysis [21]. A comprehensive analysis on presence of SSRs in *Arabidopsis* genome has been performed [22, 23]. It has been reported that the majority (80%) of all SSRs found in *Arabidopsis* genome were mono-, di-, tri-, tetra- and pentanucleotides [23]. In our analysis, maximum (22.73%) of trinucleotides were obtained in *B. juncea* compared to other 4 species studied. In *Arabidopsis* genome, SSRs in general are more favored in upstream region of the genes and trinucleotide repeated were the most common repeats found in the coding regions [22].

Comparative genomics has progressed the discovery and understanding of orthologues, but it has brought to light many fast evolving “orphan” genes of unknown function and evolutionary history. In *Brassica* species, comparative analysis provides an opportunity to study rapid genome changes associated with polyploidy level in this largest plant family. *Brassica* genome analysis might provide new insights into the organization of plant genome and the size and shape of plants as well. To accomplish this task, the complete sequence of *Brassica's* close relative, *Arabidopsis thaliana,* would be an important genomic resource.

The abundance of unigenes with cellular roles in *Brassicaceae* species was estimated by classifying the BLASTX matches with similarity to known proteins into 26 functional categories. The proportion of transcripts involved in metabolism and energy was 24.1% (between 20% and 34% among *Brassicaceae* species). Though such analysis has not been performed in case of *Brassica* species, in sugarcane assembled EST sequences with 23.8% transcripts involved in various metabolism and energy processes like bioenergetics, secondary metabolism, lipid metabolism, amino acid metabolism, DNA metabolism, nucleotide metabolism, and N, S, and P metabolism were obtained [24]. The 22% of unigenes showed similarity with that of the genes involved in storage protein, cell cycle, and DNA processes, transcription factor, protein synthesis, protein fold/modification/destination, structural/catalytic protein, protein activity regulation, and nuclear protein in different organisms. Similar types of analysis was performed in wild *Arachis stenosperma* and found that ~22% ESTs were involved in the same function [25]. Maximum numbers of unigenes analyzed in our study are still hypothetical or unknown hence could be used in functional analysis study, which may lead to discovery of some unique genes in *Brassicaceae* crops. 

PCR-based markers designed from various genomic sequences can be used for various molecular and genetic studies after their validation for quality and robustness of the amplification. Earlier reports suggest that a portion of genomic SSRs, developed in the past, have produced faint bands or stuttering [26, 27]. However, in the present study, all the genomic SSR produced clear and high-intensity bands. SSR derived from the genes have produced a high proportion of high-quality markers with strong bands and distinct alleles in most of the reports [28, 29]. The quality of genotyping data obtained from EST-SSR is highly dependent on the quality and robustness of amplification patterns. Varshney et al. [30] reported that markers derived from the conserved region of genome are expected to show greater cross-transferability between species and genera. The unigene-derived SSR markers have unique identity and positions in the transcribed region of the genome. With the availability of huge unigene databases, large-numbered SSR can be easily identified. The markers developed in present study would be an important resource for the brassica breeders. These markers would be useful for generating comparative genetic and physical maps, study of genetic diversity, marker-assisted selection, and even positional cloning of useful genes in Brassica and other related species.

## 5. Conclusions

Our analysis on the comparative analysis of *Brassicaceae* crops with *A. thaliana* confirmed a high level of nucleotide sequence conservation. Thus, a genome scale comparison of *Arabidopsis* with *Brassica* at the sequence level provides an excellent opportunity to find some agriculturally important genes, to clone and use them in breeding programmes. The average GC content of *Brassicaceae* species was between 50%–55%. The mining of SSRs showed highest percentage of mononucleotide repeats followed by di-, tri-, and tetranucleotide repeats in all of the species except *B. juncea*. A/T repeats were the prevalent mononucleotides with more than 50% in all the 5 species. The predominant class of dinucleotide repeats in all the species was AG/GA/CT/TC, maximum in *B. *rapa. The distribution of SSRs showed the abundance of mononucleotide SSRs containing 16–30 repeats while di- and trinucleotide containing 5–10 repeats. Out of the 28 functional categories, the ruling functional category of unigenes was metabolism and energy followed by structural/catalytic protein. Comparative genomics can facilitate the study of the evolution of sequences and functions of orthologous genes and also to understand diversification and adaptation. These comparative studies have contributed to analysis of complicated quantitative traits and comparisons of the organization of the chromosomes of *Brassica*. It is expected that comparative genome analysis between *Arabidopsis* and related crop species will expedite research in the more complex *Brassica* genomes. The markers developed in present study would be an important resource for the brassica breeders. These markers would be useful for generating comparative genetic and physical maps, study of genetic diversity, marker-assisted selection, and even positional cloning of useful genes in Brassica and other related species.

## Supplementary Material

Supplementary Table 1: Functional categorization of syntenic genes of five *Brassicaceae* crops. The unigene sequences of the five *Brassicaceae* species were matched with *Arabidopsis* gene sequence database at local BLAST server using BLASTN. The annotated genes were classified into 25 different functional categories based on their homology to known proteins. The predominant functional category of unigenes was metabolism and energy followed by structural/catalytic protein. Most of the unigenes of all the species were hypothetical in nature.

## Figures and Tables

**Table 1:** List of eighteen cultivars belonging to seven different species of *Brassica* used for the analysis of SSR cross transferability.

Sr. no.	Species	Genome (Chr. no.)	Cultivars
1	*Brassica rapa*	AA (*n* = 10)	*B. rapa toria* PT303
			*B. rapa toria* TL-15
			*B. rapa toria* Sangram
			*B. rapa* cv Yellow sarson Pusa Gold
			*B. rapa* cv Yellow sarson YSPB-24

2	*Brassica carinata*	BBCC (*n* = 17)	*B. carinata* Kiran
			*B. carinata* NPC-9
			*B. carinata* KC-01

3	*Brassica juncea*	AABB (*n* = 18)	*B. juncea* Pusa Agram
			*B. juncea* Bio 902
			*B. juncea* BEC1.44

4	*Brassica napus*	AACC (*n* = 19)	*B. napus* ISN-129
			*B. napus* GSL-1
			*B. napus* GSL-2

5	*Brassica oleracea*	CC (*n* = 9)	*B. oleracea italica* Palam Smridhi
			*B. oleracea botrytis* Pusa Sharad
			*B. oleracea capitata* Pusa Ageti

6	*Raphanus sativus*	(*n* = 9)	*Raphanus sativus*

**Table 2:** Summary of gene indices of different species of *Brassicaceae* family.

Species	ESTs*	Unigene	sESTs	% EST forming unigenes
*B.juncea*	235	196	176	83.40
*B. napus*	88573	6045*	5468	6.82
*B. oleracea*	20923	10281	7104	49.14
*B. rapa*	21422	8812	5546	41.14
*R. sativus*	133	94	75	70.68

Total	131286	25428	18369	

*http://www.ncbi.nlm.nih.gov/.

**Table 3:** Genome size, number of unigenes, and similarity between unigenes and the genes of *Arabidopsis. *

Species	Genome Size (Mbp)	Number of Unigenes	BLASTN homology
*B. juncea*	1068*	196	195
*B. napus*	1129–1235*	6045	5985
*B. oleracea*	599–618*	10281	10280
*B. rapa*	468–516*	8812	8812
*R. sativus*	573**	94	94

*http://www.brassica.info/information/GenomeSize.htm.

**http://radish.plantbiology.msu.edu/index.php/RadishDB:Analysis.

**Table 4:** Different types of SSR identified in the unigenes of five *Brassicaceae* crops.

Crops	Unigenes	Monomer	Dimer	Trimer	Tetramer	Pentamer	Hexamer
*B.juncea*	196	14	3	5	0	0	0
*B. napus*	6045	3211	301	250	7	1	5
*B. oleracea*	10281	1904	385	339	5	1	4
*B. rapa*	8812	1187	423	367	5	0	4
*R. sativus*	94	22	1	1	0	0	0

Total	25428	6338	1113	962	17	2	13

**Table 5:** Details of the SSR markers used for evaluation of amplification among cultivars of Brassica and cultivar of *Raphanus sativus. *

S. no.	Primer_Id^#^	Forward primer sequence (5′ → 3′)	Reverse primer sequence (5′ → 3′)	Product Size*	Anneal. temp.^$^
1	Bn_Genomic114	TGGTATTCGGGCATTGGTAT	GCAACCACACAGAAGGACAA	153	60
2	Bn_Genomic29	GAGTCTGATCCTGCCTCCAT	CTTTGTTAGCGGCTCCATGT	171	55
3	Bn_Genomic33	CTAACGACCCTTTTCCGTCA	AAACCCCACGTTCATTTTTG	194	55
4	Bo_Genomic166	GCATGCTATGTTGGGAACCT	AAGAAGACGAGCAAGGACGA	181	65
5	Bo_Genomic76	CAAGGCCAAGGTATCTGGAA	CTCTCTGACCGAGCCATCAT	160	55
6	Bo_Genomic77	GATTGAAGGCGCTTAGGAGA	ACATGCGGGATTTTGAAGAC	151	60
7	Bo_Genomic85	GAGGCGAGTACGGTTCAAGT	CCCAAATGAGGGAAAACAAA	160	65
8	Bo_Genomic90	AGCAAGAAGAGGCTCACCAA	CCGAATAGTGATGCACCAAA	199	55
9	Br_Genomic654	GGCTCTAAGCAAGTGGGACA	AAGTGCCGATGACGAAGAAG	165	60
10	Br_Genomic664	CTCTTTCCCTTTCCGGTAGC	CTCAATCGGTTCTTCTGCTG	187	55
11	Br_Genomic665	CGGCCATTCATACCATCTCT	ACAGGAGAAAGGAGCCCGTA	152	55
12	Br_Genomic674	CGAAGCAAATGAAGCAGACA	GAGGAACGGTTCAGCAAGAG	159	55
13	Br_Genomic677	TTTTACCAACGCCTTGATCG	ACCGCCTTCATCGTATTGAC	184	55
14	Br_Genomic684	TCATTCATCCGCAGCACTAC	CACGTCTGGTTGTGCGTATC	171	55
15	Br_Genomic692	AATGTTTGCAGGAGGAGAGC	ATGCAACTGAATGTGGTGGA	156	60
16	Br_Genomic697	CTCTTTACTTGCGGGAGACG	CAGCCTCCTTCACCAAGAAC	198	55
17	Br_Genomic708	TCCCCATCGACCTTTCTTCT	GGGAACCAGCATCATCAGTT	153	NA
18	Br_Genomic709	GCTTGCTTGGCTGATTTGTT	CAAGCATTCACGACATCACC	154	65
19	Br_Genomic718	CCTCGTGTCAGCGACCATA	AAGAACACATCGCCTCCTTC	178	55
20	Br_Genomic730	ACTAGGAAGAAGAAGGGAGAAACC	CCGAGCCCATTTCATTGTAT	150	55
21	Br_Genomic732	AGCATTTGCACATGTTGGTT	ATGTGCCTCGCATGTGAAT	159	55
22	Br_Genomic739	TAGGGTGAAAGGGAAGCTCA	CGCTAATAATGGCGCTAAGG	150	60
23	Br_Genomic773	GTTTCTATCGGTGCCTCGTG	TGGCCACTATGGTTTTGTCA	163	55
24	Br_Genomic777	GCACTCAGGAGAACAGAGCA	CCTGGTAAAACGTGTTGCATT	163	55
25	Br_Genomic910	AACCCCGCTGAGCTTTACTC	GTTTCCAGGAATCGCTTTGA	159	55
26	Br_Genomic935	TACCCAGTCCCAACTTGCTC	GATCTGCTGGTTGGGGATAG	192	55
27	Br_Genomic938	TTTTGTCCCTTTCCCAAATC	AGCACAAACCACACCCAAA	173	55
28	Br_Genomic939	TGAGAACAGAAGCTCAGAGTCG	TTCCTCTGTGACTGACTACAAAACA	150	NA
29	Br_Genomic940	TTTCTCCAAGGCAGAGAGGA	CACCGCATGATTTGATTGAA	178	55
30	Br_Genomic942	AGGGAGAAAACGCGTTGATA	CCTGGATGTTTGCACCCTAT	171	55
31	Br_Genomic944	ACTTGGCTCTCGATTTGCTC	TGGAGTGTTCCATCTTCCAT	176	NA
32	Br_Genomic945	CGGAACCACCTCCTTGTCTA	AACCGGTTTGTCAGGTTTTG	157	NA
33	Br_Genomic946	CGGTGAGAGAGAGGGAGATTC	TCTCCTTTGTTTGGGCTCAG	181	55
34	Br_Genomic948	AGTTCAAACCAGGTGCTGCT	GCCGGCCCTAAGATTTATGT	152	60
35	Br_Genomic952	TGAGCAGACGAAACCTGCT	AGAAGGGAGGAAGGAACGAA	168	55
36	GSS_Bn_154	CATGCTCAAAGTAGACGCAGA	GCCTTTGCTTCACAACACAA	170	55
37	GSS_Bn_313	TTCCGTCACCTAACTAGTCACC	ATATTGAGACGCCGCAGAAC	164	55
38	GSS_Bn_366	CCAAGGGGGAGTGTTATGAA	AGAGTGAAAAGGAAAACCTCCT	175	55
39	GSS_Bn_418	GAAGCAGCATCATGCCTGTA	CAAACCATAGTCAAGGCCTCA	158	55
40	GSS_Bn_423	TCAGTGTGCGTAGGAAACAAA	GGTGGGTACCTAATGGTTGG	150	NA
41	GSS_Bn_427	TTGGATCGTACTGGCCTGAT	GTCCTTGTTATGCGGCAAAG	153	55
42	GSS_Bn_432	CACATGAACAATTGCTTGGAG	TTCGTCAAGCTACCACTGGA	174	55
43	GSS_Bn_448	AGCGGAGATTGACTCAGACC	GGCTTCGTCTAAAGCCACAG	170	NA
44	GSS_Bn_450	AAGAGCAGGCAACAAATCGT	GCTTGGCGAAGTAAAAACAA	156	NA
45	GSS_Bn_464	TCCGACGCAAACTATCATCA	TCGATCACCGATGAAGTCAC	150	55
46	GSS_Bn_469	TCTCTGGTCACCCCTCTAGC	CTCCATCGAAGAAAGCCAAA	151	55
47	GSS_Bn_483	TGTTGCTGCTTCTTCGTTTG	AATCTTCACCAACGCTGCTT	158	55
48	GSS_Bn_506	GGGAAGACCCAGAAGGAAAC	AGGGAGAGGGAGAAGTGAGG	151	55
49	GSS_Bn_517	GTGGCGACTCTGGTGAAGAC	ATTCAATTCGCTTCCTCACG	161	55
50	GSS_Bn_546	AAATAGTCGCGATGCGTTTT	TCCTTTCGTTCCGACAATTC	159	55
51	GSS_Bn_549	GCCCTGCTCGTTAGAAGAAA	AGCTTGTCCCATTCCAACAC	164	60
52	GSS_Bn_552	TCCTTTCGTTCCGACAATTC	AAATAGTCGCGATGCGTTTT	165	65
53	GSS_Bn_561	TTGCCATCTCTCCTTCGATT	CTGGAGGTGCAGCTTTGACT	168	55
54	GSS_Bn_568	TCCCGTACGATCCTTTGAAC	ATGACGCGACGGATTATGA	151	55
55	GSS_Bn_570	GACAAAAAGAGCCCACATGAA	AGCAGGTTCTTCTCCACCAA	168	65
56	GSS_Bn_571	GGTTGCTACGGTGGAGCTAA	GAGGTTGAGACGGAAAAGCA	156	65
57	GSS_Bn_574	CTAATCGACGCAGACGACAG	TTTGGCTTCTCCTCGAACTC	179	55
58	GSS_Bn_577	GGCATTCTCAGGTCAGTGCT	GGTCCTGTTCCTGAATTCCTC	159	NA
59	GSS_Bn_579	CTACCACCGGGAAGAAAACA	CCCTCTGTCTCCCAGTACCA	150	55
60	GSS_Bn_583	TGGGAATTGCAACATGAAGA	CAAAGATCGGCGAAGAAGAC	190	55
61	GSS_Bn_606	CCGGATAGAGATGGAAATGG	ATTCTCCTCAGCAGCAGCA	189	55
62	GSS_Bn_612	GCTTGCATGTGCTAGGTTCA	ATACGAGCAAGGACGAGACG	183	NA
63	GSS_Bn_613	AGGAATGGGTCAGATCAAGC	GTCGCTGTCTCTCTCGTCCT	194	NA
64	GSS_Bn_614	GCCCACAAAAATGCTGAAAT	TTCGCTTGATTAGCTGGAAA	156	65
65	GSS_Bn_616	GAATCAAGCCACCAGCACTT	AGGAGTGATGAGCTGGCAGT	152	60
66	GSS_Bn_617	TCGAGTCATAACGCCTTTCG	AAACCGGTCGGTTAAAATCA	160	65
67	GSS_Bn_620	AAGGTGAAGCCTTTGGGTCT	AAGCCAACGAAAGCAAAAAC	165	55
68	GSS_Bn_622	TGTGGTGATTGCTGCTACAGA	TATGCAGCTGCTTTTGCTTC	189	NA
69	GSS_Bn_623	AAGACCCAAGACCCAAGACC	CAGCTTGGAGAGAGAGGAAGG	151	55
70	GSS_Bn_624	ATAACAGTCGTCCCCCTTCC	CAGCAGAGACTTGTGGGACA	189	55
71	GSS_Bn_625	CTTCGCCTCGATAAAGAACG	TGTTATATGGGGAAAGGTAGGC	177	55
72	GSS_Bn_626	AACCGAACCGAAAACCAAA	TGTTGCGCGGGATTATTTAT	152	55
73	GSS_Bn_628	GGGTGACCGTGATCATAGTGT	CTTCTGCATCGTCCAAATCA	167	55
74	GSS_Bn_629	CGGACCTATTCCTCAAGAGC	AGGAGACAAGGAGCCACTCA	199	55
75	U_Boleracea_320	AATCTGAATGGGCAGTTTGG	GAGCAGCCCTCATCATCATC	190	60
76	U_Boleracea_321	TGAGTCCACAAACCAGACCA	TCATCATCTTTGTTGGGACCT	162	55
77	U_Boleracea_404	AGAAGAAGAAGGGCCAAACC	TCCGAGCTTAGATCTGTCGAG	161	NA
78	U_Boleracea_433	GAACCGCTCATGAATGCTACT	CCCCAAGGATTAGGAAGTGG	200	55
79	U_Boleracea_447	CTATTCCGGCGAACTTCTCA	ACGATCCGTTACTCCCGTTA	150	55
80	U_Boleracea_460	CCCGGAAGAGTTTCCCTATC	ATCCCCTGAGAAGCTGGAAT	174	55
81	U_Boleracea_503	ATCAACACAGCCTCCAGCTT	GTTGATTCGGGTGCAAGAGT	153	55
82	U_Boleracea_504	TCCGATCAAGTCCCATCAAT	CACTGCTTCCCCTTTAGCAG	152	55
83	U_Boleracea_505	TAACCCAGGAGGTGGTCAAG	TTTGGGGTCATACCGTTTGT	171	NA
84	U_Boleracea_506	AAGGACGCTGAAGCTACCAA	TCAAGGCCGCTACCATTTAG	187	55
85	U_Boleracea_507	TGAAGAGCTTCGAAGGAAGG	ACCGTGTGAGAATCCGAAAG	175	55
86	U_Boleracea_508	TACCGGGGAAAGAAGAAGGT	TTTCAGAATCTGCAGCAACC	163	55
87	U_Brapa_244	ATCGCAGCCTCGAGATTACT	GATCGGTGAACGGATAAGGA	158	55
88	U_Brapa_421	GGCATGGCCAGCATATAAGT	CCTCCATGAGACTTCGTCAA	170	55
89	U_Brapa_422	GGCAGAAGCATCTCCTGAAG	CGCTATGGTCCCTTTTCAGT	172	55

^#^Primer Id denoted as Bn: *Brassica napus*, Bo: *Brassica oleracea*, Br: *Brassica rapa, *GSS: Genomic survey sequences, U: Unigene.

*Expected product size in base pairs.

^$^Optimized annealing temperature in °C,

NA: not amplified.
