# Supplementary material for: Comparative Analysis and EST Mining Reveals High Degree of Conservation among Five Brassicaceae Species
Source: Comp Funct Genomics. 2010 Sep 20;2010:520238. doi: 10.1155/2010/520238 (PMC2945637; doi:10.1155/2010/520238)
Supplement: Supplementary file 1 — Supplementary Table 1: Functional categorization of syntenic genes of five Brassicaceae crops. The unigene sequences of the five Brassicaceae species were matched with Arabidopsis gene sequence database at local BLAST server using BLASTN. The annotated genes were classified into 25 different functional categories based on their homology to known proteins. The predominant functional category of unigenes was metabolism and energy followed by structural/catalytic protein. Most of the unigenes of all the species were hypothetical in nature. [file 520238.f1.pdf]

Supplementary Table 1: Functional categorization of syntenic genes of five *Brassicaceae* crops. The unigene sequences of the five *Brassicaceae* species were matched with *Arabidopsis* gene sequence database at local BLAST server using BLASTN. The annotated genes were classified into 25 different functional categories based on their homology to known proteins. The predominant functional category of unigenes was metabolism and energy followed by structural/catalytic protein. Most of the unigenes of all the species were hypothetical in nature.

Function	*B.juncea*	*B.napus*	*B.oleracea*	*B.rapa*	*R. sativus*
Metabolism and Energy	60	1263	1895	1643	20
Storage Protein	1	36	119	25	1
Cell cycle and DNA processing	1	14	29	12	-
Transcription factor	1	86	198	188	-
Protein Synthesis	3	47	133	80	5
Protein fold/modifications	-	71	119	51	1
Structural/catalytic protein	16	601	1315	1222	11
Protein Activity Regulation	8	156	159	231	5
Cellular Transport	5	197	343	294	4
Cellular Communication	-	32	56	44	-
Cell Rescue, Defense and Virulence	6	177	209	139	2
Interaction with the Cellular Environment	1	13	39	22	-
Interaction with the Environment (systemic)	-	7	11	15	-
Transposable elements, Plasmid Proteins	-	2	9	2	-
Leucine zipper	1	4	5	6	-
Developmental (Systemic)	1	49	33	28	1
Biogenesis of cellular Components	1	28	138	52	2
Cell Differentiation	-	18	116	8	-
Organ Differentiation	-	4	5	2	-
Subcellular Differentiation	-	7	5	8	-
Cell Localization	11	466	295	313	20
Organ Localization	-	7	29	5	-
Ubiquitous Expression	1	59	73	68	-
Nuclear Protein	-	97	82	59	-
Hypothetical proteins	23	1045	1874	1177	6
TOTAL	140	4486	7289	5694	78
